# Socioeconomic status in patients with Turner syndrome

**DOI:** 10.1016/j.cpnec.2021.100030

**Published:** 2021-01-23

**Authors:** Iris D. Noordman, Janiëlle AEM. van der Velden, Henri JLM. Timmers, Nicole Reisch, Annette Richter-Unruh, Catherine Pienkowksi, Nel Roeleveld, Hedi L. Claahsen-van der Grinten, the dsd-LIFE group

**Affiliations:** a Department of Pediatric Endocrinology, Amalia Children’s Hospital, Radboud University Medical Center, Nijmegen, the Netherlands; b Department of Internal Medicine, Radboud University Medical Center, Nijmegen, the Netherlands; c Department of Endocrinology, Medizinische Klinik IV, Klinikum der Universität München, München, Germany; d Kinderendokrinologie und Diabetologie, Universitätsklinikum Ruhr-Universität Bochum, Kinderklinik, Bochum, Germany; e Reference Center for Rare Gynecological Pathologies, Children Hospital, Toulouse, France; f Department for Health Evidence, Radboud University Medical Center, Nijmegen, the Netherlands

**Keywords:** Turner syndrome, Socioeconomic status, Karyotype

## Abstract

**Background:**

Turner syndrome (TS) is a genetic condition with a broad phenotypic spectrum. In contrast to the medical conditions, socioeconomic factors are not well understood. Our goal was to evaluate the socioeconomic status (SES) among women with TS in a European-wide cohort, and to look for possible associated factors.

**Methods:**

This study was part of the multicenter dsd-LIFE study, including 328 women with TS. We evaluated SES (education, occupation and income) using patient-reported outcomes. Furthermore, information was collected on karyotype, age at diagnosis, comorbidity, marital status, social integration and discrimination. Reference data on SES were retrieved from the European Social Survey. Linear and logistic regression analyses were performed to compare SES of the study population with the reference population, and to analyze possible associated factors.

**Results:**

Women with TS showed a high level of education, employment status and satisfaction with income. In contrast, fewer women were living together and fewer social activities were reported compared with the reference population. The latter factors were more strongly associated with SES than medical factors. The unemployment rate was the highest in TS women aged 26–30 years, while a low education was associated with a later age at diagnosis. No major differences in SES were found among the different karyotype groups.

**Conclusions:**

The SES in women with TS was generally comparable with the reference population, although they were less frequently living with a partner or having social activities. More attention is needed for (early) psychosocial screening and support, and strategies for earlier diagnosis of TS are necessary.

## Highlights

• The socioeconomic status of women with Turner syndrome is generally not impaired.
• However, they live less often with a partner and report fewer social activities.
• These factors are more strongly associated with socioeconomic status than comorbidity.
• A low educational level is associated with a later age at diagnosis.
• Early psychosocial intervention might help to prevent transitional difficulties.

## 1 Introduction

Turner syndrome (TS) is a chromosomal condition that affects phenotypic females who have one intact X chromosome and complete or partial absence of the second sex chromosome [1]. The main well-described features are short stature, ovarian dysfunction and comorbidities such as cardiac malformations, thyroid and renal disorders. TS is also associated with a specific neurocognitive phenotype, which includes intact intellectual function and verbal abilities, but relative weaknesses in visual-spatial, executive and/or social cognitive tasks [2]. The above-mentioned features may lead to impaired quality of life in some domains, reported by several studies [3,4]. However, not much is known about socioeconomic status (SES) among patients with TS.

SES is the descriptive term for the position of persons within society, based on three main components: (1) level of education, (2) occupation, and (3) income [5]. It is well known that SES can influence the health of individuals and vice versa[6].

SES among patients with TS has been described by a number of study groups [[7], [8], [9], [10], [11]]. They have mostly reported higher levels of education compared with the general population and a comparable employment status. Gould et al. have investigated SES in 261 American women with TS, and found a baccalaureate degree or higher in 70% of these women and an employment rate of 80.4%, both higher compared with controls [8]. A large birth cohort registry study from Denmark, describing SES in 831 patients with TS, has shown that patients were more likely to retire early and had a lower income before the age of 30 [10]. Naess et al., however, have found a high degree of satisfaction with their financial and leisure situation among patients with TS [9].

Since these studies have only evaluated SES in selected cohorts with patients from one country and studies describing karyotype-phenotype associations with SES are scarce, the aim of this study was to evaluate SES in a large population of European women with TS, compared with the general European population. Furthermore, we investigated whether there is an association between SES and factors such as karyotype, age at diagnosis, comorbidity, marital status, social integration and discrimination among women with TS.

## 2 Methods

### 2.1 Study population

This study was part of the European dsd-LIFE study (https://www.dsd-life.eu/), a cross-sectional clinical outcome study, which aims to improve treatment and care of patients included under the umbrella term ‘disorder/differences of sex development’ (DSD). More detailed information about dsd-LIFE has been published earlier [12,13]. Participants aged 16 years and older with TS and other forms of DSD were recruited from February 2014 through September 2015 in fourteen study centers in six European countries: Germany, France, The Netherlands, Poland, Sweden, and The United Kingdom. From the total dsd-LIFE cohort, information of 346 patients diagnosed with TS was available. Patients with a non-classifiable karyotype (n=4) and patients with a male phenotype (n=14; 45,X/46,XY males) were excluded. The final study population consisted of 328 women with TS.

Information about the study population (women with TS) was collected in two ways. The first part (medical part) consisted of a medical interview, retrospective chart review and medical examinations. The second part of the study (patient-related outcomes (PROs)) included standardized instruments and self-constructed questionnaires. In the current study, the results of the PROs were analyzed, mainly focusing on the results of the self-reported questionnaires regarding sociodemographic data. Information on comorbidities was obtained from the first part (medical part) of the study. Written informed consent was obtained from all participants. If the participant was under the age of 18 years, both the participant and the parents signed the informed consent. Ethical approval was obtained as appropriate to each country.

### 2.2 Reference population

Reference data on sociodemographic and economic factors were retrieved from the European Social Survey (ESS, http://www.europeansocialsurvey.org), Batch 7 (2014). Using frequency matching in 5-years age groups, age-matched female participants from Germany, France, The Netherlands, Poland, Sweden, and The United Kingdom were included, in order to achieve similar age distributions between the study- and the reference population. In total, 6577 women from the same countries as the study population were eligible for inclusion. After frequency matching for age, the reference population consisted of 1911 European women.

### 2.3 Study design

This study consisted of two parts:

(1) The SES in the study population was evaluated and compared with the reference population. The SES consisted of three main components: level of education, occupational status and satisfaction with income. These factors were studied in the study- and reference population and in defined age groups (15–25, 26–50 years, and >50 years). In addition, marital status and social integration in both of the populations were evaluated, as these factors also seem to be important in the evaluation of SES [3,10]. Since the distribution of country of residence was different in the study- and reference population, all analyses were corrected for country of residence.
(2) Within the study population, the effects of possible associated factors (karyotype, age at diagnosis, comorbidity, marital status, social integration and discrimination) on the three main components of SES in women with TS were analyzed. Analyses were adjusted for age, because age strongly influences the SES.

### 2.4 Description of outcome variables

To evaluate the SES in the study population, standardized questions and classifications according to the ESS were used, which are summarized in Table 1. In addition, self-constructed questions were added to evaluate specific elements in the TS population only. Dichotomous variables were created to analyze the outcome variables. To create these dichotomous variables, one category/question was used as a ‘reference category’. First, this reference category was compared with all other answering options. Second, the other answering options were separately compared with the reference category. For the three main components of SES, this was done as follows (see Table 1):

- Level of education: Medium level of education was used as a reference category and compared with all other answering options (medium versus other). Thereafter, patients with a high level or a low level of education were compared with patients with a medium educational level (high versus medium and low versus medium)
- Occupational status: The prevalence of ‘paid work’ was used as a reference category and compared with all other answering options (paid work versus other), followed by the other answering options being separately compared with the ‘reference category’ paid work (e.g. unemployed versus paid work).
- Satisfaction with household income: ‘Living very comfortably on present income’ was used as a reference category and compared with all other answering options (living comfortably versus other). Thereafter, the other categories were compared with the ‘reference category’. The answering options ‘finding it difficult on present income’ and ‘finding it very difficult on present income’ were combined, resulting in a variable ‘finding it (very) difficult on present income’ versus the reference category.

**Table 1:** Questions and classification used to assess socioeconomic status and possible associated factors in women with TS and a European reference population.

Subject	Classification/question	Type	Answering options
Socioeconomic status			

**Level of education**	‘What is the highest level of education you have successfully completed’? → Categorized according to the ES-ISCED^§^: standardized scale that measures education in seven levels.	ESS	Answering options: ES-ISCED I – V2 Classification: • Low: ES-ISCED I and II • Medium: ES-ISCED IIIb, IIIa and IV. • High: ES-ISCED V1 or V2
	‘About how many years of education have you completed, whether full-time or part-time?’	ESS	Number of years

**Occupational status**	‘Which of these descriptions best describes your situation in the last seven days?’	ESS	Answering options • In paid work^&^ (or away temporarily; employee, self-employed, working for your family business) • In education (not paid for by employer), even if on vacation • Unemployed and actively looking for a job • Unemployed, wanting a job but not actively looking for a job • Permanently sick or disabled • Retired • In community or military service • Doing housework, looking after children or other persons • Other
	‘How many hours do you/did you usually work a week?’	ESS	Number of hours

**Satisfaction with income**	‘Which of the descriptions comes closest to how you feel about your household’s income nowadays?’	ESS	Answering options • Living very comfortably on present income^&^ • Coping on present income • Finding it difficult on present income • Finding it very difficult on present income
**Possible associated factors**			

**Subjective general health**	‘How is your health in general?’	ESS	Answering options • Very good • Good • Fair^&^ • Bad • Very bad
**Marital status**	‘How are you living’?	SC	Answering options • Single or separated, living alone • Married or in a legally registered civil union, living with partner^&^ • Living with partner without being married or in a civil union^&^ • Having a partner, but not living with him/her in the same househould • Living with parents
	‘How many children live regularly in your household?’∗	ESS	Number of children

**Social integration and discrimination**	‘Compared to other people of your age, how often would you say to take part in social activities?’	ESS	Answering options • Much more than most • More than most • About the same^&^ • Less than most • Much less than most
	‘How many people, if any, are there with whom you can discuss intimate and personal matters?’	ESS	Number of people
	‘Have you been discriminated against because of your condition?’	SC	Answering options • Yes • No
	‘Over the past 12 months, have you had contact with any support groups for your condition?’	SC	Answering options • Yes • No

ESS = European Social Survey question. SC = self-constructed question. ^§^ES-ISCED Classification: I = less than lower secondary; II = lower secondary; IIIb = lower tier upper secondary, IIIa = upper tier upper secondary, IV = advanced vocational, sub-degree; V1 = lower tertiary education, BA level; V2 = higher tertiary education, ≥ MA level. ∗including biological children, children by egg/sperm donation, stepchildren and adopted children. ^&^=this category was used as a reference category to compare the other variables with.

### 2.5 Possible associated factors

Karyotype - The study population was divided into eight subgroups based on their karyotype: 1. monosomy 45,X; 2. mosaicism 45,X/46,XX; 3. isochromosome; 4. deletion; 5. polyploidy; 6. ring X material; 7. Y-material; and 8. unknown. A monosomy 45,X karyotype was used as a reference category; all the other karyotypes were separately compared with monosomy 45,X.

Comorbidity - Comorbidity was divided into five subgroups: cardiac comorbidity (hypertension, coarctation of the aorta, bicuspid aortic valve (BAV) and aortic stenosis), renal comorbidity (horseshoe kidney, renal insufficiency and urinary tract infections), endocrinopathy (obesity, diabetes mellitus type 1, diabetes mellitus type 2, insulin resistance and Hashimoto thyroiditis), visual/auditory problems, and other comorbidities (crohn/colitis, celiac disease, fatty liver, hepatitis, hyperlipidemia, osteoporosis and fractures). Patients’ perspective on their own health was assessed with an ESS question (see Table 1). Patients reporting to have a (very) good health and a (very) bad health were compared with patients who reported to have a fair health.

Marital status and social integration - Questions regarding marital and social status are summarized in Table 1. For the analysis of marital status, one subgroup ‘Living with partner’ was created (regardless of patients being married or in a civil union or not), to be able to compare the study population with the reference population. This group was also used as a reference category for all other answering options. Patients with ‘(much) more’ and ‘(much) less’ social activities, were compared with patients who answered ‘the same as other people of my age’ (reference category).

### 2.6 Statistical analysis

Statistical analysis was performed using SPSS version 25. Descriptive statistics were used to describe baseline characteristics of the study- and the reference population. Visual inspection of e.g. skewness, kurtosis and shape of the histogram was used to determine normality of the continuous variables. The following statistical analyses were executed:

(1) To compare the SES of the study population with the reference population, binary logistic regression was used to calculate Odds Ratios (OR) with 95% confidence intervals (95%CI). Thereafter, the OR was adjusted for country of residence (OR_adj_), to correct for the differences in country of residence between the study- and the reference population. For continuous variables, linear regression was used to correct for country of residence leading to p-values from the adjusted analyses (P_adj_).
(2) To investigate associations between SES and possible associated factors within the study population, ORs and 95%CIs were calculated and corrected for age (OR_adj_). Linear regression was used to look for associations and to correct for age in continuous variables (P_adj_).

ORs were only calculated if there were at least three cases in one subgroup. P-values <0.05 and ORs with 95%CIs excluding 1 were considered statistically significant and relevant, respectively.

## 3 Results

### 3.1 Baseline characteristics of the study population

The baseline characteristics of our study population are summarized in Table 2. Monosomy 45,X was the most common karyotype (46%), followed by the presence of an isochromosome (18%), mosaicism 45,X/46,XX (10%), and Y-material (10%). The most frequently reported comorbidity was a visual and/or auditory problem.

**Table 2:** Baseline characteristics of 328 women with Turner syndrome.

Age in years	
Median (range)	28 (15–73)
Age at diagnosis in years	
Median (range)	10 (0–61)
Karyotype	
• Monosomy 45,X	150 (46%)
• Mosaicism 45,X/46,XX	31 (10%)
• Isochromosome	59 (18%)
• Deletion	19 (6%)
• Polyploidy	16 (5%)
• Ring material	12 (4%)
• Y-material	31 (10%)
• Unknown	10 (3%)
Comorbidity∗	
• Cardiac	93/320 (29%)
• Renal	48/316 (15%)
• Endocrine	128/318 (40%)
• Visual/auditory	192/321 (60%)
• Other	189/324 (58%)

### 3.2 Socioeconomic status: study population versus reference population

Table 3 shows the socioeconomic status of the study population compared with the reference population. Most women with TS lived in France (39%) and the Netherlands (25%), whereas the reference population was more uniformly distributed over the different countries of residence included in this study.

**Table 3:** Socioeconomic status of women with Turner syndrome versus a European reference population.

	Participants with Turner syndrome (n=328)	ESS reference population (n=1911 women)	P-value	OR or P-value adjusted for country of residence (OR_adj_ and P_adj_)
**Age in years** (Median (range))	28 (15-73)	29 (15-75)	P=0.906	

**Country of residence**			P<0.001	

• Germany	49 (15%)	425 (22%)		
• France	129 (39%)	272 (14%)		
• The Netherlands	83 (25%)	253 (13%)		
• Poland	6 (2%)	328 (17%)		
• Sweden	50 (15%)	286 (15%)		
• United Kingdom	11 (3%)	347 (18%)		

**Level of education**				

• High	100 (34%)	433 (23%)	P=0.013	**1.7 (1.3-2.3)**
• Medium	144 (49%)	889 (47%)	P=0.496	0.9 (0.7-1.2)
• Low	49 (17%)	569 (30%)	P<0.001	**0.6 (0.4-0.9)**

**Educational years** (median (range))	14 (1-26)	13 (1-31)	P<0.001	**P=0.009**

**Occupational status**				

• Paid work	152 (50%)	918 (48%)	P=0.528	1.1 (0.8-1.4)
• Education	82 (27%)	480 (25%)	P=0.833	1.0 (0.8-1.4)
• Unemployed	33 (11%)	144 (8%)	P=0.124	1.5 (0.9-2.3)
• **Sick/**disabled	17 (6%)	40 (2%)	P=0.001	**2.2 (1.2-4.2)**
• Retired	10 (3%)	58 (3%)	P=0.909	1.0 (0.5-2.1)
• Housework	3 (1%)	233 (12%)	P<0.001	**0.1 (0.0-0.3)**
• Other	6 (2%)	31 (2%)	P=0.731	1.0 (0.4-2.4)

**Hours worked** Median (range)	35 (0-60)	37 (0-113)	P<0.001	**P=0.008**

**Satisfaction with income**				

• Living comfortably	110 (40%)	635 (34%)	P=0.045	1.1 (0.8-1.4)
• Coping	125 (46%)	878 (47%)	P=0.163	1.0 (0.8-1.3)
• Difficult	30 (11%)	278 (15%)	P=0.029	0.8 (0.5-1.2)
• Very difficult	9 (3%)	79 (4%)	P=0.250	0.8 (0.4-1.8)

**Marital status**				

• Married/living together	100 (33%)	894 (47%)	P<0.001	**0.5 (0.4-0.7)**
• Living with parents	100 (33%)	N/A		
• Single/separated	95 (31%)	N/A		
• Having a partner, not living in the same household	9 (3%)	N/A		

Living with children	61 (20%)	753 (39%)	P<0.001	**0.4 (0.3-0.5)**

**Social activities**				

• (much) more than others	26 (9%)	299 (16%)	P=0.002	**0.5 (0.3-0.8)**
• The same as others	158 (52%)	929 (49%)	P=0.380	1.0 (0.8-1.3)
• (much) less than others	123 (40%)	677 (36%)	P=0.613	1.3 (1.0-1.7)
Discrimination based on condition	64 (21%)	N/A		
Contact with support groups	67 (22%)	N/A		

The prevalence was calculated based on available data. P_adj_ = the corrected p-value for country of residence using linear regression. OR_adj_ = the Odds Ratio adjusted for country of residence using binary logistic regression.

#### 3.2.1 Level of education

**Fig. 1:**
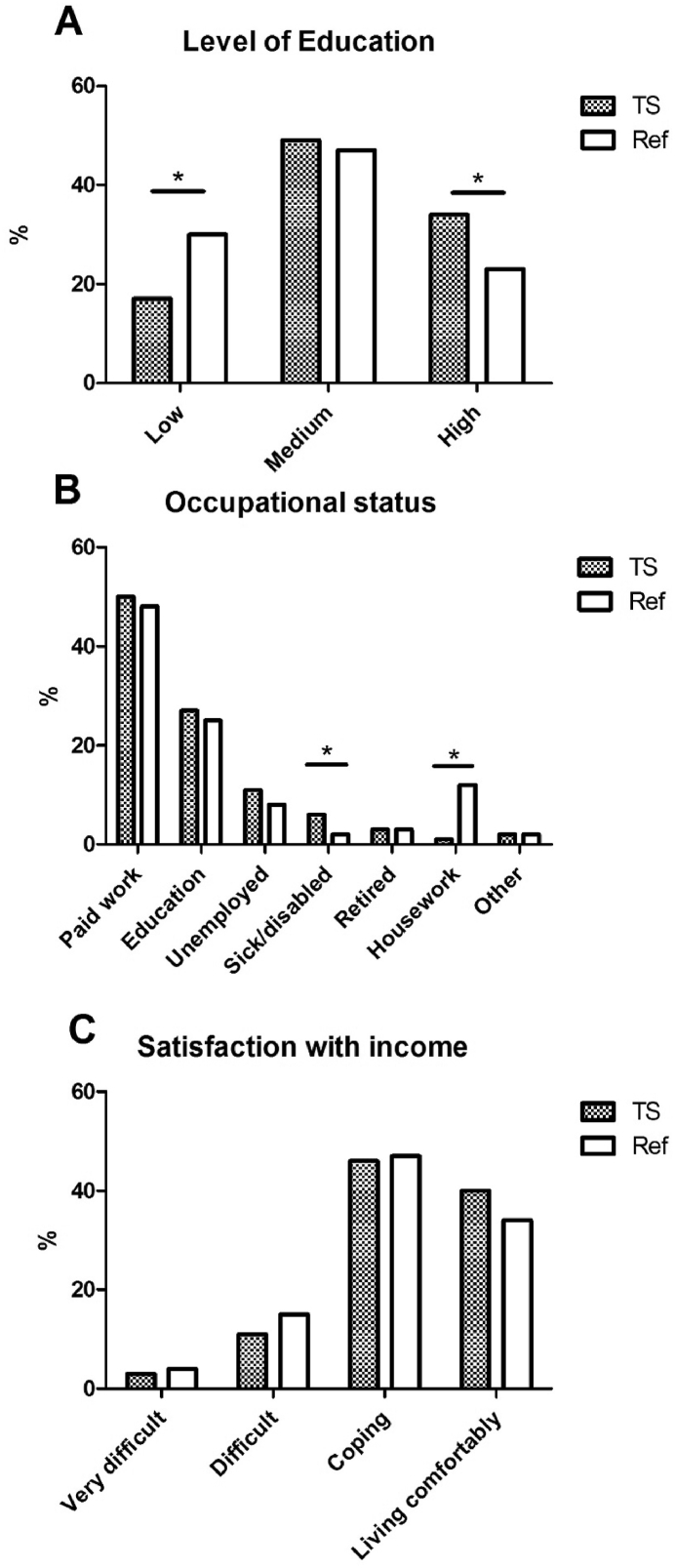
Educational level, occupational status and satisfaction with income in women with Turner syndrome compared with an age-matched European reference population. ∗OR_adj_ (95%CI) excludes 1, TS ​= ​women with Turner syndrome, Ref ​= ​European reference population.

Information on level of education was available for 293 of the 328 patients with TS. On average, patients with TS achieved a higher level of education compared with the reference population (Fig. 1, Table 3). This difference was observed in all age-groups.

#### 3.2.2 Occupational status

Information on occupational status was available for 303 of the 328 patients with TS. Women with TS more frequently reported to be sick or disabled, and less often reported to be doing housework or looking after children compared with the reference population (See Fig. 1, Table 3). The unemployment rate between the study- and the reference population did not differ significantly. However, unemployment status in women with TS aged 26–50 years was higher compared with the same age group in the reference population (OR_adj_ 95%CI: 2.4 (1.4–4.1)). Rate of unemployment was the highest among women with TS aged 26–30 (27%).

#### 3.2.3 Satisfaction with income

Information on satisfaction with household income was available for 274 of the 328 patients with TS. Most women with TS were satisfied with their present household income (‘living comfortably’ or ‘coping’, see Fig. 1, Table 3). Only nine women (3%) reported to be living very difficult on the present income. There were no differences in income satisfaction between the study population and the reference population or among different age groups within the study population itself.

#### 3.2.4 Marital status and social integration

Women with TS from all age groups were less frequently married or living together with a partner compared with the reference population. They mostly had 4-6 persons to discuss personal matters with (38% versus 40% in the reference population). Women with TS reported to have fewer social activities compared with others of their age (Table 3).

### 3.3 Socioeconomic status: associated factors within the study population

Table 4 shows the multivariate analyses of the association between SES and possible associated factors within the study population.

**Table 4:** Analyses of the main outcomes and possible associated factors in our study population of women with Turner syndrome.

	Education		Occupational status			Satisfaction with income	
	High	Low	Paid work	Unemployed	Sick/Disabled	Living comfortably	(Very) difficult
	N=100	N=49	N=152	N=33	N=17	N=110	N=39
Karyotype 45,X	0.8 (0.5–1.4)	1.0 (0.5–2.1)	0.8 (0.5–1.3)	1.7 (0.7–3.8)	2.2 (0.7–6.9)	0.7 (0.4–1.1)	1.6 (0.8–3.6)
Age at Diagnosis	p=0.506	P=0.006	p=0.063	P=0.204	P=0.08	P=0.394	P=0.305
Good subjective general health	1.8 (1.0–3.4)	0.8 (0.3–1.7)	2.3 (1.3–4.2)	0.3 (0.1–0.7)	0.2 (0.0–0.5)	3.4 (1.8–6.3)	0.3 (0.1–0.7)
Comorbidities							
• Cardiac	1.1 (0.6–1.9)	1.0 (0.5–2.3)	0.5 (0.3–0.9)	2.1 (0.9–4.8)	2.6 (0.9–7.9)	0.7 (0.4–1.1)	2.4 (1.1–5.3)
• Renal	0.8 (0.4–1.7)	2.1 (0.9–4.9)	0.6 (0.3–1.2)	1.3 (0.4–3.8)	1.7 (0.4–7.1)	0.8 (0.4–1.7)	1.2 (0.4–3.4)
• Endocrine	0.8 (0.5–1.4)	0.9 (0.5–1.8)	0.9 (0.5–1.4)	1.5 (0.7–3.3)	1.0 (0.4–3.0)	0.9 (0.5–1.5)	1.6 (0.7–3.4)
• Visual/auditory	0.8 (0.5–1.4)	1.0 (0.5–2.1)	0.9 (0.5–1.4)	1.2 (0.5–2.8)	0.6 (0.2–2.2)	1.3 (0.8–2.3)	1.3 (0.5–3.0)
• Other	0.6 (0.3–1.0)	1.0 (0.5–2.0)	0.9 (0.5–1.5)	0.9 (0.4–1.9)	1.5 (0.4–5.9)	0.8 (0.5–1.3)	1.5 (0.7–3.5)
Marital status							
• Married/living with partner	0.8 (0.5–1.5)	0.4 (0.2–1.1)	3.4 (1.9–5.9)	0.6 (0.3–1.5)	0.3 (0.1–0.9)	1.6 (0.9–2.8)	0.6 (0.2–1.3)
• Separated/single	2.1 (1.1–3.8)	1.8 (0.6–5.5)	0.5 (0.3–0.9)	1.1 (0.4–2.8)	3.2 (0.9–11.0)	0.6 (0.3–1.1)	1.6 (0.7–4.0)
• Living with parents	0.2 (0.1–0.5)	3.1 (0.8–12.6)	0.1 (0.0–0.2)	4.6 (1.1–19.1)	23.6 (1.3–419.0)	0.7 (0.3–1.7)	1.9 (0.4–8.0)
Children in household	0.7 (0.4–1.5)	0.9 (0.4–2.1)	1.2 (0.6–2.1)	0.6 (0.2–1.8)	–	2.1 (1.1–4.1)	0.3 (0.1–1.0)
Much participation in social activities	1.6 (0.6–4.2)	0.9 (0.3–3.7)	1.4 (0.6–3.3)	–	–	1.3 (0.6–3.2)	–
Experienced discrimination	0.4 (0.2–0.7)	0.4 (0.2–1.1)	1.2 (0.7–2.1)	0.7 (0.3–1.9)	1.8 (0.6–5.3)	0.5 (0.3–1.0)	2.3 (0.9–5.4)
Contact with support group	1.0 (0.5–1.9)	0.5 (0.2–1.6)	0.7 (0.4–1.2)	1.5 (0.6–3.6)	1.7 (0.6–5.2)	0.5 (0.3–1.0)	2.5 (1.1–5.9)

Values given are Odds Ratios (OR) with 95% Confidence intervals (95%CI), corrected for age using binary logistic regression (=OR_adj_), or P-values corrected for age using linear regression (=P_adj_). ORs were only calculated if a subgroup contained three or more cases.

#### 3.3.1 Level of education

Patients with high educational levels were more often separated or single, less often lived with their parents and experienced less discrimination because of their condition compared with patients with a lower level of education. In addition, the age at diagnosis was higher in patients with a low level of education compared with patients with medium level of education, even when corrected for age.

#### 3.3.2 Occupational status

In general, subjective general health and marital status were the most important factors associated with occupational status in women with TS. In regard to the comorbidities analyzed, only cardiac comorbidity was associated with a lower chance of having a paid job. No clear associations were found in the other occupational groups and were therefore not shown in Table 4.

#### 3.3.3 Satisfaction with income

Women who were satisfied with their present household income reported a better general health status. The group of women who lived with children in their household were more often satisfied with their income compared with others. Low satisfaction with income was associated with worse general health and cardiac comorbidity. Additionally, this group of women was more often in contact with a support group.

### 3.4 Possible associated factors

*Karyotype* - The SES of seven different karyotype groups is shown in Supplement A (Table 5). Karyotype monosomy 45,X was not associated with level of education, occupational status or satisfaction with income (see Table 4). Based on small numbers in some karyotype groups, not all comparisons could be made. Patients with an isochromosome were more likely to be living comfortably on their present income (OR_adj_ 95%CI: 2.5 (1.2–4.9)) compared with patients with monosomy 45,X. No other associations between karyotype and SES were found. Patients with a ring X karyotype seemed to have comparable levels of education with the other TS women and the reference population, although the rate of unemployment was relatively high (40%), and satisfaction with income relatively low. The hours worked per week and the number of educational years were similar.

*Marital status* - Being separated or single was associated with cardiac comorbidity (OR_adj_ 95%CI: 2.1 (1.1–3.9)). Furthermore, women living with children in the same household were more often married or living with a partner (OR_adj_ 95%CI: 3.2 (1.7–6.2)) compared with others.

*Social integration* - Having fewer social activities than others, was associated with worse subjective health (OR_adj_ 95%CI: 0.3 (0.2–0.5)). They experienced more discrimination (OR_adj_ 95%CI: 2.2 (1.2–3.9)) and were more often sick or disabled (OR_adj_ 95%CI: 6.7 (1.7–25.7)). Women who had contact with a support group less often had a mosaicism karyotype (OR_adj_ 95%CI: 0.2 (0.0–0.9)), reported worse subjective general health (OR_adj_ 95%CI: 0.5 (0.3–0.8)) and had more ‘other comorbidities’ (OR_adj_ 95%CI: 2.1 (1.1–4.0)).

## 4 Discussion

This study, investigating a large group of 328 individuals with TS from fourteen study centers in six European countries, shows that the socioeconomic status of European women with TS is generally not impaired compared with a reference population. Women in our study population had a high level of education, employment status and satisfaction with income. However, patients were less likely to be married or living together with a partner in all age groups and reported to have fewer social activities compared with other people of their age. Furthermore, unemployment rate in women aged 26–30 years was relatively high.

### 4.1 Level of education

In our study population, women with TS were in general highly educated. This finding is supported by other studies [[8], [9], [10], [11],^14^]. However, the definition of ‘high educational level’ differs among studies and countries. In this study, the ESS criteria were used. This made it possible to compare the educational levels of the study population with the European population. The high employment status could possibly be explained by academic accommodations which have expanded over the last years. A recent study has investigated cognitive deficits, such as visuospatial deficits, and provision of academic accommodations, such as extra time or remedial classes, in females with TS [15]. Their results showed progress in the obtainment of academic accommodations needed within recent decades for females with TS. Furthermore, coping skills of this patient group, including perseverance in the face of adversity and equability of temperament [8], could be an explanation for the achievement of a high educational attainment.

A low educational status was associated with later age at diagnosis in our study. Reimann et al. has reported that patients with a late diagnosis (≥13 years), were more likely to develop depressive symptoms and decreased deception of competence [16]. These observations suggest that an early diagnosis may contribute to a higher educational status, besides the well-known advantages like an early start of growth hormone treatment, puberty induction and timely screening for associated comorbidities [17]. Earlier detection of problems, such as hearing difficulties, neurocognitive problems and social difficulties, could lead to personalized counseling and treatment, creating the optimal environment for the child to develop in school and at home. With improvement of knowledge and diagnostics, such as screening for TS in girls with short stature, patients could be diagnosed earlier. More studies are needed to evaluate the exact mechanisms behind the associations between level of education and factors like age at diagnosis.

### 4.2 Occupational status

In the literature, a wide range of employment rate has been described (29%–90%), probably explained by the different age-ranges of the study populations [[7], [8], [9],^11^,^14^,^18^,^19^]. As expected, fewer patients had a paid job in the younger populations, and more patients were in education. Gould et al. have found a higher prevalence of patients with paid work compared to controls (80% versus 70%), whereas our study and most other studies have shown comparable rates in patients with TS versus controls. Some even described that patients with TS have lower occupational status than would be expected from the level of education, and that they have fewer positive/challenging working experiences [20,21]. This specific topic was not investigated in the current study.

Patients with TS more often reported to be sick or disabled compared with controls, especially in the patient group aged 50 or higher, most likely due to a higher morbidity rate in patients with TS [22]. Although one study has described retirement at an earlier age compared with controls [10], this was not observed in our (relatively young) study population[22].

The prevalence of unemployment in the total TS population was comparable with the prevalence in the reference population. However, a higher unemployment rate in women with TS aged 26–50 years was found, with the highest prevalence among women aged 26–30 years. We hypothesize that girls with TS do well in the protected environment of education, but face more difficulties when looking for or starting a new job.

### 4.3 Satisfaction with income

European women with TS are similarly satisfied with their income as the reference population. Income in women with TS has only been described by two studies in the past. In a cohort of 80 women with TS, Naess et al. have found that they were more satisfied with their income compared with controls [9]. In contrast, a Danish registry study has reported that the proportion of women with TS with an income below the median was increased before the age of 30 years, and was similar with controls thereafter [10]. In our study, no difference in satisfaction with income among age groups was found, although the difference in marital status between the study population and reference population has to be taken into account.

The association between income and subjective general health in patients with TS was also found in the reference population and has been described by others [23]. An explanation for the association between satisfaction with income and children in the household may be the high costs of assisted reproductive technology (e.g. egg donation or in vitro fertilization) and adoption procedures in many countries[24].

### 4.4 SES and associated factors

In general, comorbidities were not associated with SES, except for cardiac comorbidities. Even visual- and auditory problems were not associated with SES in our study. This might indicate that comorbidities are adequately treated and have a low impact on SES in patients with TS. Marital status and social factors, however, were more clearly associated with SES in patients with TS in our study.

Recent studies have described that patients with TS are less ready for (medical) transition than age-matched patients with other chronic conditions, and that they lack some of the transition readiness skills [25,26]. Besides these medical transition difficulties, it seems that (young) women with TS also struggle with other transitions in life, including difficulties in the transition from school to a working life, but also moving out from their parents’ house or starting a romantic relationship. This hypothesis is supported by the relatively high unemployment rate in the age group 26–30 years in our study, the high prevalence of women living with their parents and the low prevalence of women being married or living with a partner in all different age groups. The decreased self-esteem, which has been frequently described in patients with TS, might play a role in these transitional problems [3,18,21]. Early screening for psychosocial problems, as suggested in the clinical TS guideline, may lead to better psychosocial support, better self-esteem and in the end less (negative) impact on SES[1].

The TS guideline also advises to encourage early involvement of patients in TS support groups. Our data suggest that patients who contacted a support group in the past twelve months, had more medical and social problems. This probably explains the association between karyotype and contact with a support group, as patients with monosomy 45,X/46,XX are known to have a less severe phenotype compared with other karyotypes [12]. The value of a support group has never been investigated in patients with TS, but studies investigating other diseases have shown that a support group may be of additional value for patients [27,28]. Patients should be encouraged to join TS support groups, which are now contacted by a minority, but could play an important role in the psychosocial support for girls and women with TS.

### 4.5 SES and karyotype

No major differences in SES were found between the different karyotype groups. Patients with an isochromosome karyotype seemed to be more satisfied with their income compared to patients with monosomy 45,X. We have no clear explanation for this association, although we have shown in a previous study that patients with an isochromosome karyotype have a less severe phenotype compared with monosomy 45,X [12]. In our study population, patients with ring X seemed to show comparable educational levels to the other karyotypes and the reference population. This is an interesting finding, as other studies have reported a higher prevalence of mental retardation and cognitive impairment in the ring X group. Kuntsi et al. have described a group of 47 patients with a ring X chromosome, and have found an increased risk of learning difficulties and associated behavioral maladjustment [29]. In the latter study, 63% of the patients with a ring X chromosome had special educational needs and only 69% had been to a mainstream school, whereas the patients in our study showed educational levels comparable with the other karyotypes and the reference population. However, our study population included only twelve patients with this karyotype. SES in patients with a ring X karyotype has been described by only one study before, which also showed that SES in this group was similar to the other karyotype groups[8].

### 4.6 Limitations

Despite the large study population and the well described reference group, several limitations apply to this study. Most women with TS were recruited from patient support groups and outpatient clinics with the risk of selection bias. It might be that women with TS with a higher educational level were more willing to participate in this study, more integrated in follow up in specialized centers, and more willing to complete the rather long questionnaire. Some of the questions from the questionnaire were self-constructed and not validated, although most of the questions used in this study were ESS questions. This allowed us to compare our study population with a European reference population. As for the variable satisfaction with income, it is important to note that this is a subjective measure, not necessarily reflecting the actual income. Last, there were some missing data, which are reported in the tables. Small numbers, especially in the karyotype groups, require the results to be interpreted with caution.

## 5 Conclusion and recommendations

SES in patients with TS is generally not impaired. Patients with TS had higher levels of education and comparable employment status and satisfaction with income compared with the general European population. In contrast, patients with TS were less likely to be living together with a partner and reported fewer social activities. The unemployment rate in women aged 26–30 years was relatively high.

The most important factors associated with SES were subjective general health, social difficulties and marital status. Comorbidities appeared to have less impact on SES. A low level of education was associated with a later age at diagnosis, so strategies to allow for early diagnosis of TS are necessary.

In general, transitions seem to be difficult for women with TS, including the transition from school to working life, but also moving out from their parents’ house or starting a romantic relationship. Apart from early diagnosis and medical intervention, early psychosociological intervention should be developed to reinforce and support the individual’s self-esteem. In addition, contact with a support group might help to prevent difficulties with transitions and should be encouraged.

## Declaration of competing interest

The authors declare that they have no known competing financial interests or personal relationships that could have appeared to influence the work reported in this paper.
