# Supplementary material for: Socioeconomic status in patients with Turner syndrome
Source: Compr Psychoneuroendocrinol. 2021 Jan 23;5:100030. doi: 10.1016/j.cpnec.2021.100030 (PMC9216711; doi:10.1016/j.cpnec.2021.100030)
Supplement: Multimedia component 1 [file mmc1.docx]

# Supplement A

| Table 5: Socioeconomic status in 7 different karyotype groups of women with Turner syndrome. | | | | | | | |
| --- | --- | --- | --- | --- | --- | --- | --- |
|  | **Monosomy**  **45,X^&^** | **Mosaicism**  **45,X/46,XX** | **Isochromo-some** | **Deletion** | **Polyploidy** | **Ring X** | **Y-material** |
|  | N=150 | N=31 | N=59 | N=19 | N=16 | N=12 | N=31 |
| Education |  |  |  |  |  |  |  |
| High | 43 (33%) | 10 (39%) | 17 (30%) | 6 (38%) | 6 (43%) | 5 (46%) | 8 (29%) |
| Medium | 68 (52%) | 12 (46%) | 29 (52%) | 6 (38%) | 7 (50%) | 5 (46%) | 13 (46%) |
| Low | 21 (16%) | 4 (15%) | 10 (18%) | 4 (25%) | 1 (7%) | 1 (9%) | 7 (25%) |
| Occupation |  |  |  |  |  |  |  |
| Paid Work | 68 (49%) | 13 (48%) | 27 (48%) | 10 (63%) | 9 (60%) | 3 (30%) | 15 (52%) |
| Education | 34 (24%) | 12 (44%) | 22 (39%) | 3 (19%) | 3 (20%) | 2 (20%) | 6 (21%) |
| Unemployed | 17 (12%) | 0 | 3 (5%) | 2 (13%) | 1 (7%) | 4 (40%) | 3 (10%) |
| Sick/disabled | 12 (9%) | 1 (4%) | 0 | 0 | 0 | 0 | 4 (14%) |
| Retired | 7 (5%) | 0 | 0 | 1 (6%) | 2 (13%) | 0 | 0 |
| Housework | 1 (1%) | 0 | 1 (2%) | 0 | 0 | 0 | 1 (3%) |
| Other | 1 (1%) | 1 (4%) | 3 (5%) | 0 | 0 | 1 (10%) | 0 |
| Income |  |  |  |  |  |  |  |
| Comfortably | 41 (33%) | 7 (33%) | 28 (56%) | 4 (27%) | 8 (57%) | 3 (27%) | 12 (43%) |
| Coping | 62 (50%) | 12 (57%) | 17 (34%) | 9 (60%) | 4 (29%) | 5 (46%) | 13 (46%) |
| Difficult | 17 (14%) | 0 | 5 (10%) | 2 (13%) | 2 (14%) | 1 (9%) | 3 (11%) |
| Very difficult | 5 (4%) | 2 (10%) | 0 | 0 | 0 | 2 (18%) | 0 |
| Values are given as n (%). ^&^Monosomy 45,X was used as a reference category; all other karyotype groups were separately compared with monosomy 45,X karyotype | | | | | | | |
